# Inhibitory Effect of Statins on Inflammation-Related Pathways in Human Abdominal Aortic Aneurysm Tissue

**DOI:** 10.3390/ijms160511213

**Published:** 2015-05-18

**Authors:** Koichi Yoshimura, Ayako Nagasawa, Junichi Kudo, Masahiko Onoda, Noriyasu Morikage, Akira Furutani, Hiroki Aoki, Kimikazu Hamano

**Affiliations:** 1 Department of Surgery and Clinical Science, Yamaguchi University Graduate School of Medicine, Ube 755-8505, Japan; E-Mails: nagasawa.ayako@gmail.com (A.N.); junkud@yb3.so-net.ne.jp (J.K.); m-onoda0911@cap.ocn.ne.jp (M.O.); morikage@yamaguchi-u.ac.jp (N.M.); akirafut@a011.broada.jp (A.F.); kimikazu@yamaguchi-u.ac.jp (K.H.); 2 Graduate School of Health and Welfare, Yamaguchi Prefectural University, Yamaguchi 753-8502, Japan; 3 Division of Thoracic and Cardiovascular Surgery, Niigata University Graduate School of Medical and Dental Science, Niigata 951-8510, Japan; 4 Cardiovascular Research Institute, Kurume University, Kurume 830-0011, Japan; E-Mail: haoki@med.kurume-u.ac.jp

**Keywords:** statin, abdominal aortic aneurysm, nuclear factor-κB

## Abstract

HMG-CoA (3-hydroxy-3-methylglutaryl-coenzyme A) reductase inhibitors (statins) have been suggested to attenuate abdominal aortic aneurysm (AAA) growth. However, the effects of statins in human AAA tissues are not fully elucidated. The aim of this study was to investigate the direct effects of statins on proinflammatory molecules in human AAA walls in *ex vivo* culture. Simvastatin strongly inhibited the activation of nuclear factor (NF)-κB induced by tumor necrosis factor (TNF)-α in human AAA walls, but showed little effect on c-jun *N*-terminal kinase (JNK) activation. Simvastatin, as well as pitavastatin significantly reduced the secretion of matrix metalloproteinase (MMP)-9, monocyte chemoattractant protein (MCP)-2 and epithelial neutrophil-activating peptide (CXCL5) under both basal and TNF-α-stimulated conditions. Similar to statins, the Rac1 inhibitor NSC23766 significantly inhibited the activation of NF-κB, accompanied by a decreased secretion of MMP-9, MCP-2 and CXCL5. Moreover, the effect of simvastatin and the JNK inhibitor SP600125 was additive in inhibiting the secretion of MMP-9, MCP-2 and CXCL5. These findings indicate that statins preferentially inhibit the Rac1/NF-κB pathway to suppress MMP-9 and chemokine secretion in human AAA, suggesting a mechanism for the potential effect of statins in attenuating AAA progression.

## 1. Introduction

Abdominal aortic aneurysm (AAA), which is a segmental dilatation of the abdominal aorta, is a common and life-threatening disease, especially in older men [1,2]. AAA is characterized by chemokine-directed infiltration of inflammatory cells and destruction of extracellular matrix by proteolytic enzymes, such as matrix metalloproteinases (MMPs), eventually leading to fatal rupture [3,4,5,6]. We and others demonstrated previously that signaling pathways, such as c-jun *N*-terminal kinase (JNK) and nuclear factor (NF)-κB, play a key role in regulating the expression of proinflammatory mediators, including cytokines, and in activating MMPs in AAA and that inhibiting these signals is highly effective in treating experimental AAA [7,8,9]. However, despite advances in understanding the molecular pathogenesis of AAA, non-surgical treatment options have not been available for these patients. Especially, pharmacotherapies for AAA have been long awaited [5,10,11,12].

The 3-hydroxy-3-methylglutaryl-coenzyme A (HMG-CoA) reductase inhibitors (known as statins) are a well-established class of cholesterol-lowering drugs reported to be beneficial in various vascular diseases, because of their pleiotropic effects [13,14,15,16]. Based on *ex vivo* culture analyses, statins reduce the secretion of proinflammatory proteins, including MMP-9 and monocyte chemoattractant protein (MCP)-1, in human AAA walls [17,18,19]. Treatment with statins also confers protection against AAA development in animal models [20,21,22,23], and several clinical studies have shown an association between statin administration and decreased AAA growth [24,25,26,27]. However, the therapeutic effect of statins in AAA patients remains controversial [28,29,30,31,32]. In addition, the mechanism of action for statins in human AAA tissues has not been fully elucidated, although a better understanding of this mechanism is essential for taking advantage of statins in treating patients with AAA.

The aim of this study was to elucidate the direct effects of statins on proinflammatory molecules in human AAA tissues. Using an *ex vivo* culture system, we show that statins primarily inhibit the NF-κB pathway to suppress the secretion of chemokines and MMP-9 in human AAA walls.

## 2. Results and Discussion

### 2.1. Effect of Simvastatin on JNK and NF-κB Activation in Human AAA Walls

**Figure 1:**
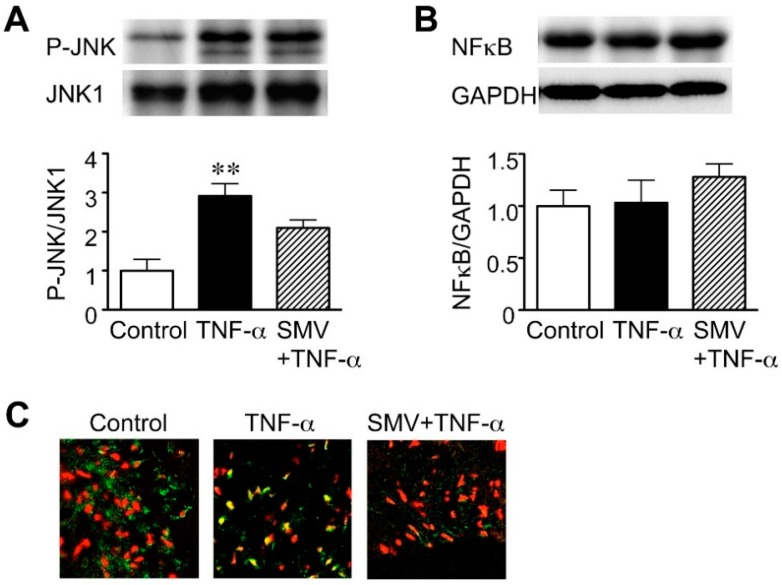
Effect of statins on c-Jun *N*-terminal kinase (JNK) and nuclear factor (NF)-κB activation in human abdominal aortic aneurysm (AAA) wall. A human AAA wall was cut into small pieces and cultured with tumor necrosis factor (TNF)-α (50 ng/mL), simvastatin (SMV, 10 μM) or vehicle (control). (**A**) Levels of phosphorylated JNK (P-JNK) and JNK1 in the cultured tissues were determined by Western blot. Representative results and quantitative analysis are shown (*n* = 4). Data are the mean ± standard deviation. ** *p* < 0.01 compared to the control; (**B**) Levels of NF-κB in the cultured tissues were determined by Western blot. Glyceraldehyde-3-phosphate dehydrogenase (GAPDH) was used as an internal control. Representative results and quantitative analysis are shown (*n* = 4); (**C**) Nuclear translocation of NF-κB in cultured tissues was analyzed by immunofluorescence staining. Representative results are shown for NF-κB (green) and cell nuclei (red). Yellow indicates overlapping localization of red and green signals.

We first examined whether statins can act on the JNK and NF-κB signaling pathways, both of which are believed to be key proinflammatory signaling pathways in the pathogenesis of AAA [7,8,9]. For this purpose, we analyzed the phosphorylation of JNK and nuclear translocation of NF-κB. As a stimulus, we used tumor necrosis factor (TNF)-α, because it is elevated in both serum and aneurysm walls of patients with AAA [33,34] and implicated in AAA pathogenesis [35]. Stimulation of cultured human AAA walls with TNF-α (50 ng/mL) caused a large increase in the phosphorylation of JNK (2.9-fold, *p* = 0.0051 compared to the control; Figure 1A), indicating JNK activation without any change in the total expression level of JNK1. Simvastatin (10 μM) attenuated JNK phosphorylation (28% reduction, compared to TNF-α; Figure 1A), but this effect did not reach statistical significance. Although TNF-α stimulation did not change NF-κB protein expression levels (Figure 1B), TNF-α led to nuclear translocation of NF-κB, indicating NF-κB activation (Figure 1C). Interestingly, treatment with simvastatin apparently suppressed the TNF-α-induced nuclear translocation of NF-κB (Figure 1C). These data suggest that statins preferentially inhibit the activation of NF-κB rather than of JNK in human AAA walls.

### 2.2. Effect of Statins in Downstream Pathways after NF-κB

**Figure 2:**
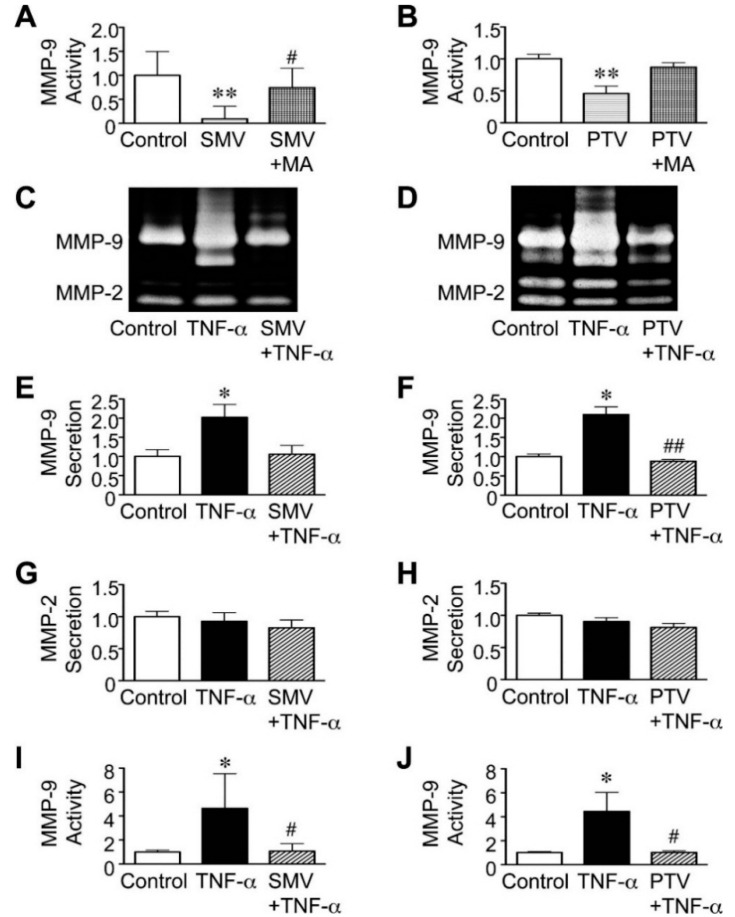
Effect of statins on matrix metalloproteinase (MMP)-9 secretion in human abdominal aortic aneurysm (AAA) wall. A human AAA wall was cut into small pieces and cultured with simvastatin (SMV, 10 μM), pitavastatin (PTV, 20 μM), mevalonate (MA, 100 μM), tumor necrosis factor (TNF)-α (50 ng/mL) or vehicle (control). (**A**,**B**) Levels of MMP-9 activity in the conditioned media were analyzed (*n* = 5). Data are the mean ± standard deviation (SD). ** *p* < 0.01 compared to the control; ^#^ *p* < 0.05 compared to SMV; (**C**–**H**) Levels of MMP-9 and MMP-2 in the conditioned media were determined by zymography (*n* = 5). Data are the mean ± SD. * *p* < 0.05 compared to the control; ^##^ *p* < 0.01 compared to TNF-α; (**I**,**J**) Levels of MMP-9 activity in the conditioned media were analyzed (*n* = 5). Data are the mean ± SD. * *p* < 0.05 compared to the control; ^#^ *p* < 0.05 compared to TNF-α.

We next investigated the effects of statins on the effector molecules that may act in pathways downstream from NF-κB in human AAA. MMP-9 is a key molecule for the degradation of extracellular matrix in AAA walls [36] and is upregulated by NF-κB and JNK signals [7,8]. Similar to previous reports [17,19], we observed that simvastatin (10 μM), as well as pitavastatin (20 μM) reduced MMP-9 activity in human AAA walls in the basal condition, possibly in a mevalonate pathway-dependent manner (Figure 2A,B). Both statins significantly suppressed the secretion and activity of MMP-9, even after TNF-α stimulation (Figure 2C–F,I,J), while neither affected the secretion of MMP-2 (Figure 2C,D,G,H). From another perspective, these results also suggested that the viability of human AAA tissues was preserved in *ex vivo* cultures during the experiments.

**Figure 3:**
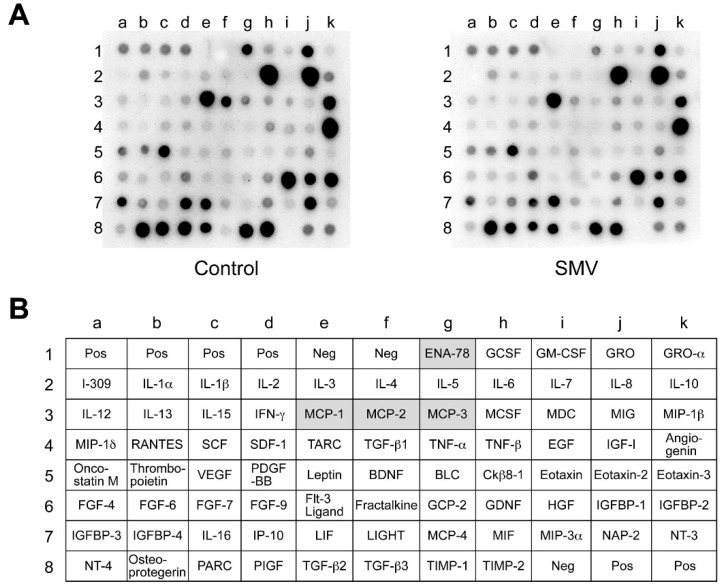
Array analysis of cytokines after statin treatment in human abdominal aortic aneurysm (AAA) wall. A human AAA wall was cut into small pieces and cultured with simvastatin (SMV, 10 μM) or vehicle (control). The conditioned media were analyzed using a cytokine array. (**A**) Representative images are shown for array membranes; (**B**) the position of cytokines and controls on the membrane are listed. Highlighted cells indicate the cytokines downregulated after SMV treatment. Pos, positive control; Neg, negative control.

Because of an earlier suggestion that NF-κB regulates gene expression of some cytokines in cultured vascular cells [37], we investigated the effect of statins on the secretion of cytokines from human AAA walls. Using a cytokine antibody array, we performed a comprehensive analysis of 79 cytokines and chemokines in the conditioned media of human AAA culture after treatment with or without simvastatin (10 μM). The array data suggested that simvastatin attenuated secretion levels of MCP-1 (CCL2), MCP-2 (CCL8), MCP-3 (CCL-7) and CXCL5 (ENA-78) (Figure 3A,B). MCP-1, -2 and -3 and CXCL5 are characterized by recruitment of monocytes and neutrophils, respectively, and have been reported to be upregulated in AAA [19,38,39,40], suggesting the involvement of these chemokines in AAA pathogenesis [6]. Simvastatin seemed to have a more potent effect on MCP-2 and CXCL5 than on MCP-1 and -3 (Figure 3A,B).

**Figure 4:**
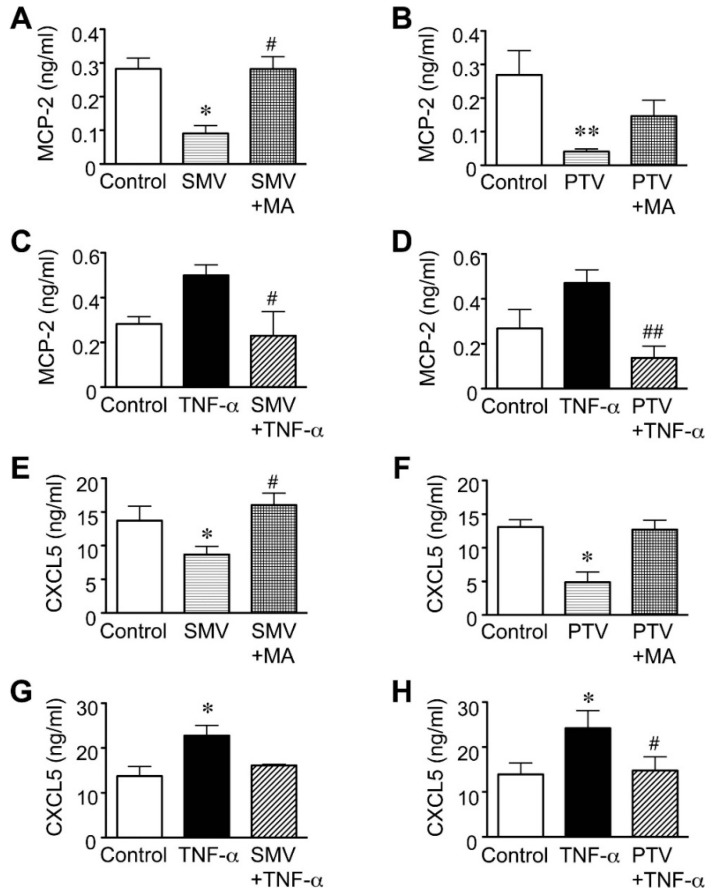
Effect of statins on chemokine secretion in human abdominal aortic aneurysm (AAA) wall. A human AAA wall was cut into small pieces and cultured with simvastatin (SMV, 10 μM), pitavastatin (PTV, 20 μM), mevalonate (MA, 100 μM), tumor necrosis factor (TNF)-α (50 ng/mL) or vehicle (control). (**A**–**D**) Levels of monocyte chemoattractant protein (MCP)-2 secretion in the conditioned media were analyzed (*n* = 5). Data are the mean ± standard deviation (SD). * *p* < 0.05 and ** *p* < 0.01 compared to the control; ^#^ *p* < 0.05 compared to SMV or TNF-α; ^##^ *p* < 0.01 compared to TNF-α; (**E**–**H**) Levels of CXCL5 secretion in the conditioned media were analyzed (*n* = 5). Data are the mean ± SD. * *p* < 0.05 compared to the control; ^#^ *p* < 0.05 compared to SMV or TNF-α.

In addition, we previously reported that simvastatin suppressed the secretion of MCP-1 and -3 under both basal and TNF-α-stimulated conditions [19]. Therefore, we focused here on quantitatively examining the effects of statins on MCP-2 and CXCL5 using the enzyme-linked immunosorbent assay (ELISA). Simvastatin (10 μM), as well as pitavastatin (20 μM) significantly reduced the secretion of MCP-2 from AAA tissues under basal condition (68% reduction, *p* = 0.0427, and 85% reduction, *p* = 0.0071, for simvastatin and pitavastatin, respectively; Figure 4A,B). Both statins also significantly suppressed the MCP-2 secretion induced by TNF-α (Figure 4C,D). Similar to their influence on MCP-2, simvastatin and pitavastatin significantly reduced the secretion of CXCL5 under the basal condition (32% reduction, *p* = 0.0473, and 63% reduction, *p* = 0.0427, for simvastatin and pitavastatin, respectively; Figure 4E,F) and tended to suppress CXCL5 secretion even after TNF-α stimulation (Figure 4G,H). Moreover, the addition of mevalonate tended to reverse the effects of both statins on the secretion of MCP-2 and CXCL5, suggesting that statins affect the levels of these chemokines in a mevalonate pathway-dependent manner (Figure 4A,B,E,F).

### 2.3. Importance of the Rac1/NF-κB Pathway Inhibition in the Action of Statins

**Figure 5:**
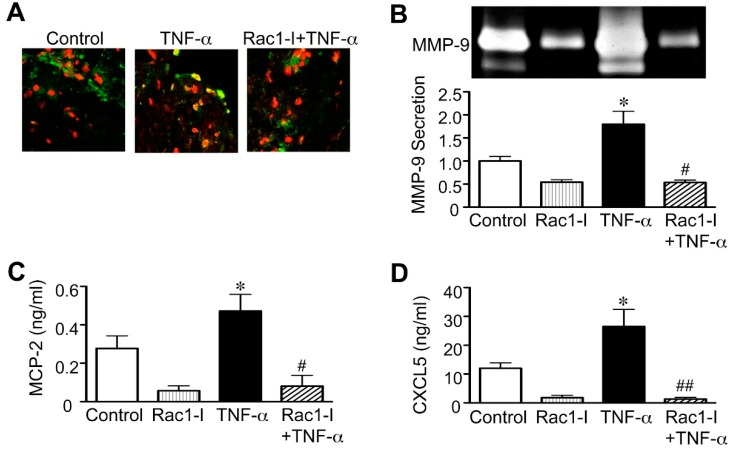
Effect of Rac1 inhibitor on human abdominal aortic aneurysm (AAA) wall. A human AAA wall was cut into small pieces and cultured with the Rac1 inhibitor NSC23766 (Rac1-I, 100 μM), tumor necrosis factor (TNF)-α (50 ng/mL) or vehicle (control). (**A**) Nuclear translocation of nuclear factor (NF)-κB in cultured tissues was analyzed by immunofluorescence staining. Representative results are shown for NF-κB (green) and cell nuclei (red). Yellow indicates overlapping localization of the red and green signals; (**B**–**D**) Levels of matrix metalloproteinase (MMP)-9, monocyte chemoattractant protein (MCP)-2 and epithelial neutrophil-activating peptide (CXCL5) in the conditioned media were analyzed (*n* = 4). Data are the mean ± standard deviation. * *p* < 0.05 compared to the control; ^#^ *p* < 0.05 and ^##^ *p* < 0.01 compared to TNF-α.

Previously, it was reported that statins suppress the activity of Rac1, a member of the Rho GTPase family [41], and that Rac1 is required for NF-κB activation in vascular cells, such as vascular smooth muscle cells and monocytes [42,43]. For this reason, we applied the Rac1 inhibitor NSC23766 to cultured human AAA walls to examine the relevance of Rac1/NF-κB pathway inhibition to the effects of statins on MMP-9, MCP-2 and CXCL5. We found that similar to statins, NSC23766 completely stopped the nuclear translocation of NF-κB that TNF-α stimulation induced (Figure 5A). NSC23766 also reduced MMP-9 secretion in human AAA walls after TNF-α stimulation (62% reduction, *p* = 0.0227; Figure 5B). Moreover, NSC23766 reduced the secretion of MCP-2 and CXCL5 in human AAA walls after TNF-α stimulation (83% reduction, *p* = 0.0109, and 95% reduction, *p* = 0.0065, for MCP-2 and CXCL5, respectively; Figure 5C,D). These findings may suggest that statins act on MMP-9, MCP-2 and CXCL5 through the Rac1/NF-κB pathway in human AAA walls.

**Figure 6:**
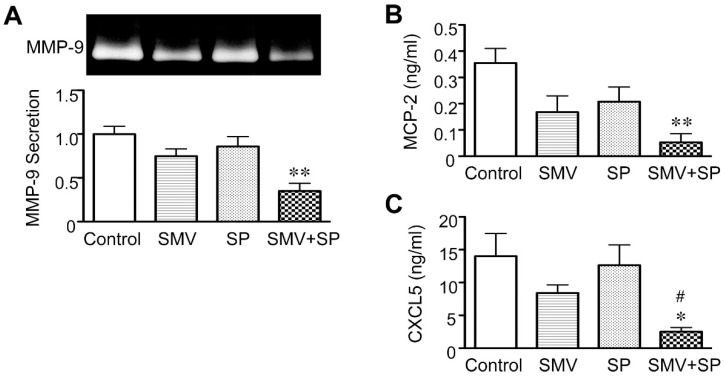
Additive effect of statin and c-jun *N*-terminal kinase (JNK) inhibitor on human abdominal aortic aneurysm (AAA) wall. A human AAA wall was cut into small pieces and cultured with simvastatin (SMV, 3 μM), JNK inhibitor SP600125 (SP, 5 μM) or vehicle (control). (**A**–**C**) Levels of matrix metalloproteinase (MMP)-9, monocyte chemoattractant protein (MCP)-2 and epithelial neutrophil-activating peptide (CXCL5) in the conditioned media were analyzed (*n* = 4). Data are the mean ± standard deviation. * *p* < 0.05 and ** *p* < 0.01 compared to the control; ^#^ *p* < 0.05 compared to SP.

Finally, to elucidate the significance of statin inhibition of Rac1/NF-κB in human AAA, we examined the combined effects of statin and JNK inhibitor on the secretion of MMP-9, MCP-2 and CXCL5. For this experiment, we used simvastatin and the JNK inhibitor SP600125 at relatively low concentrations (3 and 5 μM, respectively). Although we previously demonstrated that 50 μM SP600125 dramatically reduced MMP-9 secreted from cultured human AAA walls [7,44], the 5 μM concentration of SP600125 had little effect (Figure 6A). The 3 μM simvastatin also had little effect on MMP-9 secretion (Figure 6A). Interestingly, the combination of simvastatin (3 μM) and SP600125 (5 μM) remarkably reduced MMP-9 secretion (65% reduction compared to control, *p* = 0.0050; Figure 6A), indicating an additive effect of simvastatin and SP600125 on MMP-9 secretion. This combination also significantly reduced MCP-2 secretion (85% reduction compared to control, *p* = 0.0022; Figure 6B) and CXCL5 secretion (82% reduction compared to control, *p* = 0.0109; Figure 6C). These data suggested that the Rac1/NF-κB and JNK pathways work in parallel to cause the secretion of MMP-9, MCP-2 and CXCL5 in human AAA walls.

### 2.4. Clinical Implications and Future Directions

**Figure 7:**
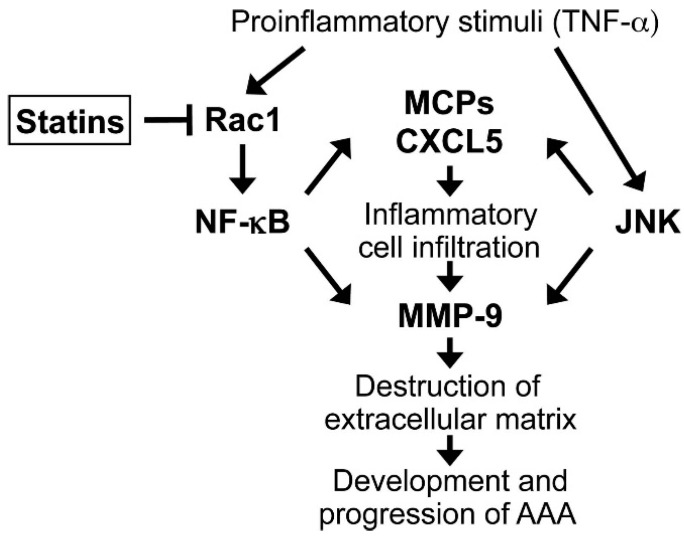
Scheme of how inhibiting the Rac1/nuclear factor (NF)-κB pathway with statins could limit abdominal aortic aneurysm (AAA) growth. Activation of both NF-κB and c-jun *N*-terminal kinase (JNK) signals results in inflammatory cell infiltration by upregulating chemokine secretion and in aortic wall destruction by activating matrix-degrading proteases. Statins preferentially inhibit the Rac1/NF-κB pathway, potentially leading to reduced progression of AAA.

Whether statins reduce AAA growth remains in question because of conflicting association evidence and the obstacles to examining the effects of statins on AAA expansion in a randomized controlled trial. The present study has revealed that treatment of human AAA tissues with statins markedly suppresses the activation of NF-κB and secretion of chemokines and MMP-9, with little effect on JNK activity. Our results indicate that statins and possibly their combination with the JNK inhibitor have the potential to reduce inflammatory cell infiltration, extracellular matrix degradation and progression of human AAA (Figure 7). However, of note, the statin concentrations employed in our experiments (e.g., simvastatin, 10 μM) were much higher than those achieved in therapeutic use (peak plasma concentration of simvastatin, 19–81 nM) [45,46]. A recent report asserted that most of the available evidence for the proposed pleiotropic effects of statins, which is based on *in vitro* studies, may be potentially misleading, because of the high statin concentrations used [46]. In addition, clinical use of statins at higher doses could lead to increased toxicity and adverse effects, such as myopathy and an asymptomatic increase in hepatic transaminases [45]. Intriguingly, previous clinical studies have reported an association between statin prescription and reduced levels in AAA walls of proinflammatory molecules, including cytokines, MMPs, NF-κB and JNK [47,48,49,50,51]. However, considering our results, these reports could be interpreted as suggesting that these anti-inflammatory effects of a statin prescription were merely secondary to a lipid-lowering effect. In practical terms, an association of statin prescription and reduced AAA growth is still controversial [30,31,52]. The chance therefore seems limited at this time that statin administration can directly reduce disease activity by attenuating proinflammatory molecules in AAA tissues or dramatically contribute to a reduction in AAA growth.

Because AAA is mostly localized to a limited site on the aorta, targeted delivery of the drugs is a reasonable approach to increase therapeutic efficacy and reduce systemic side effects [5]. If targeted drug delivery enables accumulation of statins into AAA tissues, there is an exciting possibility of taking advantage of their pharmacologic action to treat patients with AAA. Indeed, a therapeutic strategy with targeted delivery of statins has been successfully used to treat vascular lesions in animal models [53,54,55]. Further progress with targeted delivery of statins could advance the development of pharmacotherapies for patients with small AAA and for inoperable patients. In addition, the combination of local statin delivery with endovascular stent grafting is another possibility. Because ongoing AAA wall degeneration and failure of AAA exclusion are major concerns after endovascular repair and stent-graft devices can readily serve as drug-delivery platforms, adjuvant pharmacotherapy would provide an ideal solution [5,56]. To introduce statin therapy for AAA into practical use, biomarkers must be developed that enable monitoring of disease activity and tissue response during therapy to optimize the therapeutic regimen [5,10]. Fortuitously, this study has identified not only MMP-9, but also MCP-2 and CXCL5 as potential biomarkers for statin therapy for AAA, because they all showed an appreciable reduction in secretion levels with statin treatment. Although more studies will be necessary to test their potential clinical utility, these candidates or a combination of such candidates might be useful for predicting clinical responses to statins and for identifying responders and nonresponders to statin therapy among patients with AAA.

## 3. Experimental Section

### 3.1. Organ Culture of Human AAA Walls

AAA wall specimens were obtained from 17 patients at the time of open surgical repair at Yamaguchi University Hospital. The mean patient age was 74 ± 11 years. All patients were males and had a smoking history. The prevalence rates were: hypertension, 88.2%; dyslipidemia, 23.5%; and diabetes mellitus, 11.8%. The mean AAA diameter was 65 ± 16 mm. Patients taking statins and patients diagnosed with inflammatory, infectious, familial or ruptured AAA were excluded from this study (Table S1).

Organ *ex vivo* culture was performed using a method described previously [7,57,58]. Briefly, AAA wall specimens were minced into approximately 1 mm pieces. Equal wet weights of the minced tissue were placed in each well of 6-well plates and cultured with serum-free Dulbecco’s Modified Eagle’s Medium (Invitrogen, Carlsbad, CA, USA) supplemented with penicillin and streptomycin in an atmosphere of 95% air/5% CO_2_ at 37 °C. The cultured AAA walls were treated with or without simvastatin (Merck Biosciences, Nottingham, UK) or pitavastatin (Santa Cruz Biotechnology, Dallas, TX, USA) at the indicated concentrations. In some experiments, 50 ng/mL of TNF-α (R&D Systems, Minneapolis, MN, USA) was added after pretreatment with either vehicle or statin. The tissue samples of cultured AAA walls were collected 30 min after TNF-α treatment and snap-frozen in liquid nitrogen for protein extraction. The tissues were also frozen with Tissue-Tek OCT compound (Sakura Finetek, Tokyo, Japan) for immunohistochemistry. In addition, the conditioned media between 48 and 96 h after treatment were collected for the ELISA assay and gelatin zymography. When indicated, AAA tissues were treated with 100 μM mevalonate (Sigma-Aldrich, St. Louis, MO, USA), 100 μM Rac1 inhibitor NSC23766 (Calbiochem, San Diego, CA, USA) or 5 μM JNK inhibitor SP600125 (Tocris Bioscience, Bristol, UK). All experimental protocols using human specimens were approved by the Institutional Review Board at Yamaguchi University Hospital. Informed consent was obtained from all patients.

### 3.2. Protein Analyses of Tissue Homogenates and Conditioned Media

The tissue samples of the cultured AAA walls were homogenized in 25 mM Tris, pH 7.4, containing 150 mM NaCl, 5 mM EDTA, 10 mM sodium pyrophosphate, 10 mM β-glycerophosphate, 1 mM Na_3_VO_4_, 1 mM phenylmethane sulfonyl fluoride and 10 μg/mL aprotinin, and then Triton X-100 was added to a final concentration of 1% to extract proteins. Protein concentrations were determined using the BCA protein assay kit (Bio-Rad, Hercules, CA, USA). Western blotting was performed by a method described previously [7,16]. Briefly, equal amounts of sample proteins were loaded onto individual lanes in sodium dodecyl sulfate (SDS) polyacrylamide gels, separated by electrophoresis and transferred onto polyvinylidene difluoride membranes (Millipore, Billerica, MA, USA). Membranes were probed with antibodies against phospho-JNK (Promega, Madison, WI, USA, #V7931), JNK1 (Santa Cruz Biotechnology, #sc-474), NF-κB (Santa Cruz Biotechnology, #sc-8008) and glyceraldehyde-3-phosphate dehydrogenase (Millipore, #MAB374) and visualized with the use of a chemiluminescence detection kit (Thermo Scientific, Rockford, IL, USA) (Figure S1). 

Immunofluorescence staining was performed as described previously [59]. NF-κB was detected with an anti-NF-κB antibody (Santa Cruz Biotechnology, #sc-8008) using frozen sections and visualized by indirect immunofluorescence staining with the Alexa Fluor 488 conjugated antibody (Molecular Probes, Eugene, OR, USA). TOTO-3 (Molecular Probes) was used for nuclear staining. 

Gelatin zymography was performed to analyze MMP-9 and MMP-2 in the conditioned media, as described previously [7,16]. Briefly, equal volumes of conditioned media were electrophoresed in the presence of 0.2% SDS in 10% polyacrylamide gels containing gelatin (1 mg/mL) under nonreducing conditions. After electrophoresis, the gels were washed in 2.5% Triton-X 100, followed by overnight incubation at 37 °C in developing buffer containing 50 mM Tris, pH 7.5, 200 mM NaCl, 5 mM CaCl_2_ and 0.02% Brij 35. Then, the gels were stained with 0.5% Coomassie brilliant blue R-250 in 40% methanol and 10% acetic acid. The expression levels of MMP-2 and -9 were determined by quantifying clear bands corresponding in size (92 kDa, pro-MMP-9; 88 kDa, activated MMP-9; 72 kDa, pro-MMP-2; 66 kDa, activated MMP-2). Activity of MMP-9 in the conditioned media was also determined by first immunocapturing MMP-9 on the assay plate and then applying chromogenic substrate using the MMP-9 Activity Assay System (Amersham Biosciences, Piscataway, NJ, USA, #RPN2634). 

Several cytokines and chemokines in the conditioned media were detected using the Cytokine Antibody Array kit (RayBiotech, Norcross, GA, USA, #H0108005). The concentration of MCP-2 or ENA-78/CXCL5 in the conditioned media was quantified using the Human MCP-2 ELISA Kit (RayBiotech, #ELH-MCP2-001) or Human ENA-78 (CXCL5) Immunoassay kit (R&D Systems, #DX000), according to the manufacturer’s instructions. ELISA and MMP-9 activity analyses were performed by a researcher blinded to the treatment assignment, but other analyses were not.

### 3.3. Statistical Analysis

All data are expressed as the mean ± standard deviation of at least four independent experiments. To compare three or more groups, the Kruskal–Wallis test followed by Dunn’s *post hoc* test was used. Statistical analyses were performed with Prism 6.0d software (GraphPad Software, La Jolla, CA, USA).

## 4. Conclusions

In conclusion, we demonstrated the beneficial effects of statins in cultured human AAA walls, especially inhibition of the NF-κB pathway. Our results provide a mechanism for the direct effects of statins on human AAA tissues and raise the possibility that statin therapy for AAA will draw renewed attention when an appropriate drug delivery system is developed.

## Supplementary Materials

Supplementary materials can be found at http://www.mdpi.com/1422-0067/16/05/11213/s1.
