# Supplementary material for: Inhibitory Effect of Statins on Inflammation-Related Pathways in Human Abdominal Aortic Aneurysm Tissue
Source: Int J Mol Sci. 2015 May 18;16(5):11213–28. doi: 10.3390/ijms160511213 (PMC4463697; doi:10.3390/ijms160511213)
Supplement: Supplementary file 1 [file ijms-16-11213-s001.pdf]

Supplementary Information

Figure 1A

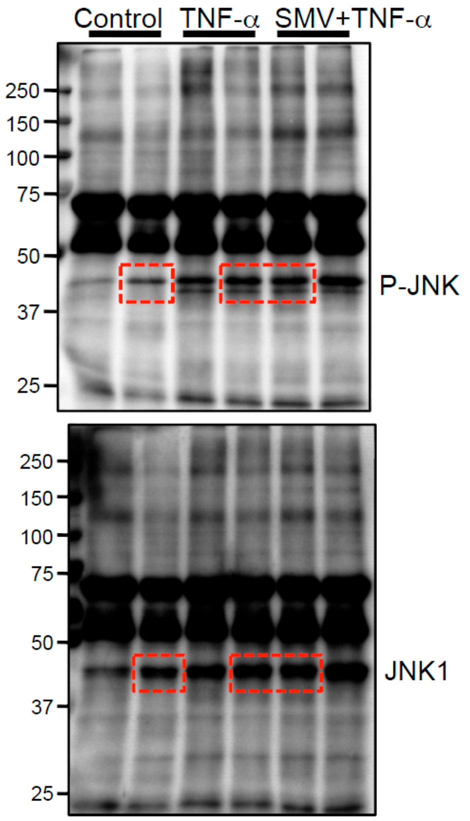

Figure 1B

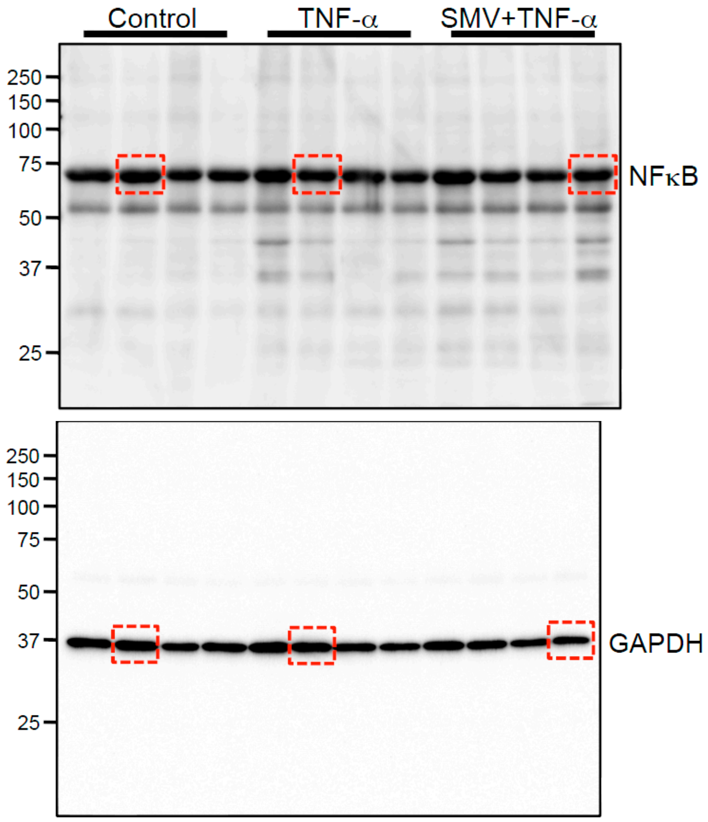

Figure 2C

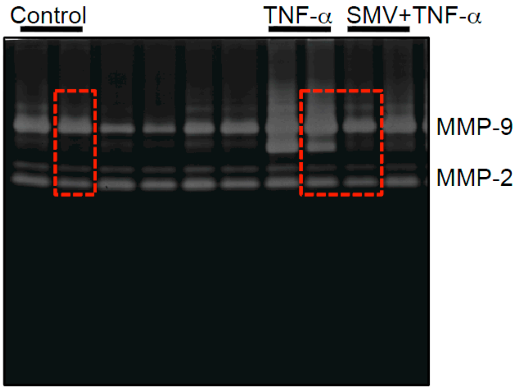

Figure 2D

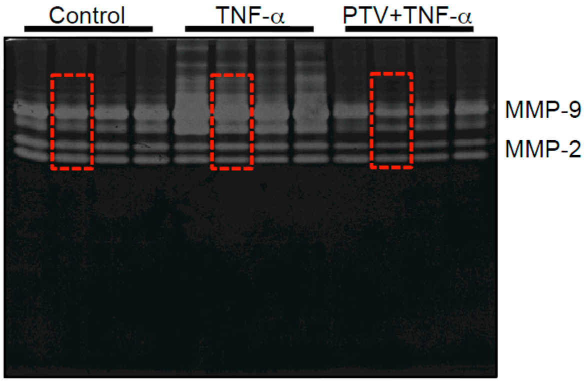

Figure 5B

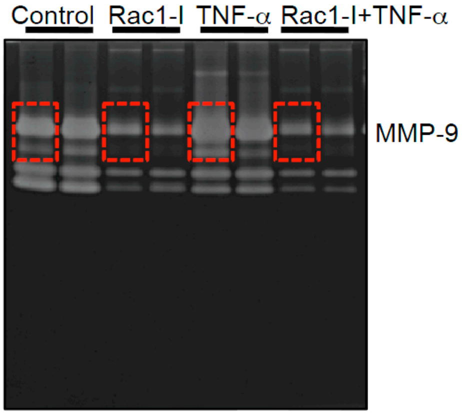

Figure 6A

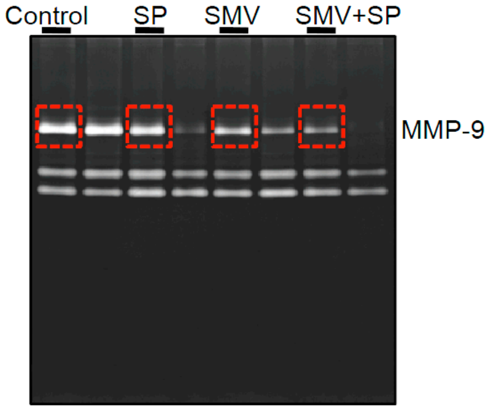

Figure S1. Uncropped full-length pictures of western blotting membranes and zymography gels.

Table S1. Clinical characteristics of patients.

No.	Age	Sex	HT	DL	DM	Sm	Statin	AAA Diameter	Allocation to experiments
1	75	M	Yes	No	No	Yes	No	50	Figures 1 and 3
2	56	M	Yes	No	No	Yes	No	49	Figures 1 and 3
3	77	M	Yes	No	Yes	Yes	No	76	Figures 1 and 3
4	72	M	Yes	No	No	Yes	No	79	Figures 1 and 3
5	86	M	Yes	No	No	Yes	No	55	Figures 2 and 4
6	76	M	Yes	No	No	Yes	No	58	Figures 2 and 4
7	88	M	Yes	No	No	Yes	No	58	Figures 2 and 4
8	68	M	Yes	Yes	No	Yes	No	90	Figures 2 and 4
9	91	M	Yes	No	No	Yes	No	50	Figures 2 and 4
10	58	M	Yes	Yes	Yes	Yes	No	51	Figure 5
11	63	M	No	Yes	No	Yes	No	83	Figure 5
12	79	M	No	No	No	Yes	No	65	Figure 5
13	55	M	Yes	No	No	Yes	No	80	Figure 5
14	76	M	Yes	No	No	Yes	No	48	Figure 6
15	80	M	Yes	No	No	Yes	No	52	Figure 6
16	86	M	Yes	No	No	Yes	No	65	Figure 6
17	72	M	Yes	No	No	Yes	No	95	Figure 6

M, male; HT, hypertension; DL, dyslipidemia; DM, diabetes mellitus; Sm, smoking; AAA, abdominal aortic aneurysm.
